# Shoulder Surgery Under Regional Anesthesia With Sedation Without General Anesthesia: A Case Series

**DOI:** 10.7759/cureus.108190

**Published:** 2026-05-03

**Authors:** Gabriel José Redondano Oliveira, Daphne Nicoletti, Adriano Marchetto

**Affiliations:** 1 Anesthesiology and Perioperative Medicine, Hospital Vera Cruz, Campinas, BRA; 2 Orthopedics, Instituto Wilson Mello, Campinas, BRA; 3 Orthopedics, Hospital Vera Cruz, Campinas, BRA

**Keywords:** sedation, shoulder surgery, superficial cervical plexus, superior trunk block, ultrasound-guided regional anesthesia

## Abstract

Introduction: In Brazil, shoulder surgeries are traditionally performed under general anesthesia combined with regional blocks. This study aimed to describe the feasibility and safety profile of regional anesthesia with sedation, without general anesthesia, for shoulder surgery.

Materials and methods: Following approval by the institutional ethics committee, 10 consecutive patients undergoing elective shoulder surgery (arthroscopy, n = 6; arthroplasty, n = 4) received an ultrasound-guided superior trunk block of the brachial plexus combined with an intermediate superficial cervical plexus block using ropivacaine. The primary outcome was the conversion rate to general anesthesia. Secondary outcomes included intraoperative hemodynamic stability, pain scores, post-anesthesia recovery time, length of hospital stay, patient satisfaction (net promoter score, NPS), and complications within 14 days.

Results: All blocks were successfully performed (100%). No patient required conversion to general anesthesia (0%). Intraoperative hypotension occurred in 30% of cases and was more frequent in arthroplasty procedures (50%) than in arthroscopy procedures (16.7%). The mean post-anesthesia care unit stay was 51 ± 10 minutes. Seventy percent of patients were discharged on the same day (83.3% of arthroscopy patients and 50% of arthroplasty patients). Only one patient (10%) required rescue opioid (tramadol) within the first 24 hours. Two patients experienced transient hoarseness, which resolved completely within nine hours. No cases of Horner syndrome, persistent neurological symptoms at 24 hours, 7 days, or 14 days, readmissions, or reoperations within 30 days were observed. The NPS was 10 in all patients.

Conclusions: Regional anesthesia alone using a superior trunk block combined with a superficial cervical plexus block is feasible and safe for shoulder surgery without the need for conversion to general anesthesia. These preliminary findings support the potential for practice change in Brazil; however, larger studies are needed to confirm these results.

## Introduction

Shoulder surgeries, including arthroscopy and arthroplasty, represent a significant proportion of orthopedic procedures and are associated with specific anesthetic challenges related to adequate pain control, intraoperative clinical stability, and postoperative recovery [1]. In Brazil, general anesthesia has been widely used for these procedures, often combined with regional blocks for analgesia. Although this combination provides excellent surgical conditions and postoperative pain control, it exposes patients to the risks and adverse effects of both techniques, including postoperative nausea and vomiting, respiratory depression, and prolonged recovery time associated with opioid use and general anesthesia, as well as hemidiaphragmatic paralysis related to regional anesthesia [2,3].

Cohen-Balaloum et al. demonstrated that regional anesthesia alone was associated with significantly reduced intraoperative hypotension (13% vs 57%), shorter post-anesthetic care unit length of stay (22 vs 52 minutes), and higher same-day discharge rates (96% vs 75%) compared with combined general and regional anesthesia in 482 patients undergoing shoulder arthroscopy [1]. Multiple studies have confirmed high success rates for regional-only approaches, with conversion rates to general anesthesia as low as 0.5-2% when ultrasound guidance is used [4,5].

The superior trunk block has emerged as an attractive alternative to the traditional interscalene block, providing non-inferior analgesia while significantly reducing hemidiaphragmatic paralysis [6,7]. Kim et al. demonstrated that a superior trunk block resulted in only a 4.8% incidence of hemidiaphragmatic paralysis compared to 71.4% with an interscalene block, while maintaining equivalent surgical anesthesia and pain control [7]. The technique involves ultrasound-guided injection around the superior trunk of the brachial plexus, formed by the union of the C5 and C6 nerve roots [6,7]. In this study, we used an out-of-plane approach, avoiding the in-plane technique, due to better sonoanatomic visualization and to avoid the theoretical risk of injury to the long thoracic and dorsal scapular nerves, which would be in the needle trajectory when traversing the middle scalene muscle. When combined with a superficial cervical plexus block to anesthetize the skin incision sites, this approach can provide complete surgical anesthesia for shoulder procedures [8].

Despite compelling international evidence, regional anesthesia alone has not been adopted in Brazilian shoulder surgery practice. To our knowledge, no published data describe the feasibility of this approach in the Brazilian healthcare setting. The objective of this prospective case series was to describe the feasibility, safety profile, and preliminary clinical outcomes of the superior trunk block combined with the superficial cervical plexus block as the sole anesthesia for shoulder arthroscopy and arthroplasty in a Brazilian surgical center.

## Materials and methods

This observational, descriptive case series study with prospective data collection was conducted at Hospital Vera Cruz between February and April 2026 following approval from the Research Ethics Committee of the Roberto Rocha Brito Foundation (approval number: 95563826.7.0000.0410). Written informed consent was obtained from all patients. The study followed the PROCESS 2023 guidelines for reporting case series in surgery [9-11].

Patient selection

Patients aged 14 years or older with American Society of Anesthesiologists (ASA) physical status classification I-III [12], scheduled for elective shoulder arthroscopy or arthroplasty, who consented to regional anesthesia alone, and with complete clinical and anesthetic records available, were eligible for inclusion [4]. Exclusion criteria comprised patient refusal; contraindications to regional anesthesia (coagulopathy, infection at injection site, or allergy to local anesthetics); inability to communicate or cooperate during surgery; severe anxiety or claustrophobia; severe pulmonary disease (FEV1 50% predicted or baseline oxygen dependence); incomplete data precluding outcome analysis; prior planning of general anesthesia from procedure onset; and emergency surgeries.

Anesthetic technique

Patients undergoing arthroscopy were positioned in the lateral decubitus position. In contrast, those undergoing arthroplasty were placed in the beach-chair position, with the head maintained in a neutral position and supplemental oxygen delivered via nasal cannula. Sedation was achieved using target-controlled infusion of propofol (Eleveld model), titrated for patient comfort while maintaining verbal communication. Ultrasound guidance was performed using a linear transducer (SonoSite®). For the superior trunk block, the ultrasound transducer was positioned in the supraclavicular fossa, scanning the brachial plexus to the interscalene groove, with identification of the superior trunk (C5-C6 root fusion). Using an out-of-plane technique with a Stimuplex A 50 mm® needle, and after negative aspiration, 11-14 mL of 0.5% ropivacaine was injected around the superior trunk. Circumferential spread was confirmed on ultrasound. Recent evidence suggests that lower volumes (5-10 mL of 0.25-0.5% ropivacaine) reduce hemidiaphragmatic paralysis while maintaining analgesic efficacy. Standard volumes described in the literature are 15 mL of 0.5% ropivacaine. For the superficial cervical plexus block, ultrasound-guided identification of the sternocleidomastoid muscle was performed with a linear transducer, followed by injection of 5-7 mL of 0.375% ropivacaine between the investing layer of the deep cervical fascia and the prevertebral fascia using a Stimuplex A 50 mm® needle. Sensory block was assessed by temperature differential in C5-C6 dermatomes at 15 and 20 minutes after block placement. Surgery was initiated when an adequate sensory block was confirmed.

Intraoperative management

Standard monitoring included continuous electrocardiography, non-invasive blood pressure measurement, and pulse oximetry. Supplemental oxygen was provided via nasal cannula throughout the procedure. Conversion to general anesthesia was defined as the need for airway management or patient request due to inadequate anesthesia.

Postoperative management

Standard post-anesthesia care unit (PACU) monitoring was performed in all patients. Multimodal analgesia consisted of dipyrone and ketoprofen, with tramadol available for rescue analgesia. Patients were considered eligible for discharge when they achieved a modified Aldrete score of 9 or higher [13].

Outcomes assessed

The primary outcome was the conversion rate to general anesthesia, reflecting the technique's feasibility. Secondary intraoperative outcomes included block success rate (defined as an adequate sensory block), surgical duration, intraoperative hypotension (mean arterial pressure <65 mmHg for >5 minutes), vasopressor requirement, desaturation, and bradycardia. Secondary postoperative outcomes included pain scores at PACU arrival on the numeric rating scale (0-10), rescue opioid requirement within 24 hours, PACU length of stay (minutes), same-day discharge rate, and patient satisfaction measured by the Net Promoter Score (NPS). Safety outcomes included hoarseness (indicative of recurrent laryngeal nerve block), Horner syndrome, neurological symptoms at 24 hours, 7 days, and 14 days, unplanned readmission, and reoperation within 30 days.

Statistical analysis

Data were collected prospectively using standardized case report forms. Continuous variables are presented as mean ± standard deviation, and categorical variables are presented as frequencies and percentages. Given the small sample size and descriptive nature of this case series, no hypothesis testing was performed.

## Results

Ten consecutive patients were included in this case series between February and April 2026, with six undergoing arthroscopy and four undergoing shoulder arthroplasty (Table 1).

**Table 1:** Baseline characteristics stratified by surgery type SD: standard deviation, BMI: body mass index, ASA: American Society of Anesthesiologists

Variable	Total (n = 10)	Arthroscopy (n = 6)	Arthroplasty (n = 4)
Age (years), mean ± SD	66.2 ± 20.8	58.7 ± 20.5	77.5 ± 17.5
Female sex, n (%)	7 (70%)	4 (66.7%)	3 (75%)
BMI (kg/m²), mean ± SD	26.6 ± 5.0	24.8 ± 2.8	29.2 ± 7.2
ASA I, n (%)	2 (20%)	2 (33.3%)	0 (0%)
ASA II, n (%)	4 (40%)	2 (33.3%)	2 (50%)
ASA III, n (%)	4 (40%)	2 (33.3%)	2 (50%)

Primary outcome

No patient (0/10, 0%) required conversion to general anesthesia, demonstrating the feasibility of the technique for both shoulder arthroscopy and arthroplasty.

Intraoperative outcomes

All blocks were performed successfully under ultrasound guidance, with a 100% success rate (10/10) (Table 2).

**Table 2:** Intraoperative outcomes stratified by surgery type GA: general anesthesia, SD: standard deviation

Variable	Total (n = 10)	Arthroscopy (n = 6)	Arthroplasty (n = 4)
Block success, n (%)	10 (100%)	6 (100%)	4 (100%)
Conversion to GA, n (%)	0 (0%)	0 (0%)	0 (0%)
Surgical duration (min), mean ± SD	-	92.7 ± 13.7	176.5 ± 41.9
Intraoperative hypotension, n (%)	3 (30%)	1 (16.7%)	2 (50%)
Vasopressor use, n (%)	3 (30%)	1 (16.7%)	2 (50%)
Desaturation, n (%)	0 (0%)	0 (0%)	0 (0%)
Bradycardia, n (%)	0 (0%)	0 (0%)	0 (0%)

The mean surgical duration was 92.7 ± 13.7 minutes for arthroscopies and 176.5 ± 41.9 minutes for arthroplasties. Intraoperative hypotension occurred in 3 patients (30%), being more frequent in arthroplasties (50%) compared to arthroscopies (16.7%). All hypotensive episodes were treated with vasopressors, without the need for conversion to general anesthesia. There were no episodes of desaturation or bradycardia.

Postoperative outcomes

The mean PACU length of stay was 51 ± 10 minutes, being shorter in arthroscopies (42.8 ± 2.5 minutes) compared to arthroplasties (63.3 ± 5.8 minutes) (Table 3). All patients met discharge criteria (modified Aldrete-Kroulik score ≥9) within this timeframe. All patients had pain scores of 0 on arrival in the PACU, demonstrating excellent immediate analgesia from the regional block.

**Table 3:** Postoperative outcomes stratified by surgery type PACU: post-anesthesia care unit, LOS: length of stay, NRS: numeric rating scale, NPS: net promoter score, SD: standard deviation

Variable	Total (n = 10)	Arthroscopy (n = 6)	Arthroplasty (n = 4)
PACU LOS (min), mean ± SD	51 ± 10	42.8 ± 2.5	63.3 ± 5.8
Pain at PACU (NRS 0–10)	0	0	0
Rescue opioid (24h), n (%)	1 (10%)	0 (0%)	1 (25%)
NPS (0-10)	10	10	10
Same-day discharge, n (%)	7 (70%)	5 (83.3%)	2 (50%)
Unplanned readmission, n (%)	0 (0%)	0 (0%)	0 (0%)
Reoperation (30 days), n (%)	0 (0%)	0 (0%)	0 (0%)

Only one patient (10%), from the arthroplasty group, required rescue opioid (tramadol) within the first 24 hours, indicating effective and prolonged analgesia from the regional block technique. No patient in the arthroscopy group required opioid rescue.

Seventy percent of patients (7/10) were discharged on the same day as the procedure, with higher ambulatory discharge rates for arthroscopies (83.3%) than for arthroplasties (50%). Admission of arthroplasty patients was related to surgical procedure complexity rather than anesthetic complications.

Safety outcomes

Two patients (20%) experienced transient hoarseness, likely related to superficial cervical plexus block with spread to the recurrent laryngeal nerve (Table 4). Both cases resolved spontaneously, one at three hours and another at nine hours, without the need for intervention. No patient developed Horner syndrome. No symptoms suggestive of clinically significant hemidiaphragmatic paralysis were observed.

**Table 4:** Safety outcomes

Variable	Total (n = 10)
Transient hoarseness, n (%)	2 (20%)
Time to hoarseness resolution	3 h and 9 h
Horner syndrome, n (%)	0 (0%)
Neurological symptoms at 24 h, n (%)	0 (0%)
Neurological symptoms at 7 days, n (%)	0 (0%)
Neurological symptoms at 14 days, n (%)	0 (0%)

At structured follow-up at 24 hours, 7 days, and 14 days, no patient presented persistent neurological symptoms, including paraesthesiae, weakness, or sensory deficits. There were no unplanned readmissions or reoperations within 30 days.

Patient satisfaction

All patients had an NPS of 10, indicating high satisfaction with the anesthetic technique. Notably, transient hoarseness did not adversely affect satisfaction among affected patients.

## Discussion

Main findings

This prospective case series demonstrates that a superior trunk block combined with a superficial cervical plexus block as the sole anesthesia is feasible and safe for shoulder arthroscopy and arthroplasty in a Brazilian surgical center. The conversion rate to general anesthesia was 0%, with a 100% block success rate, excellent postoperative pain control (pain score 0 at PACU), minimal opioid requirements (only 10% required rescue opioid within 24 hours), a short recovery time (51 minutes), and a high ambulatory procedure rate (70%). Notably, all patients reported maximum satisfaction (NPS 10) with the anesthetic technique.

Opioid-sparing effect

The low rescue opioid requirement observed in this series (10% overall, limited to one arthroplasty patient) highlights the opioid-sparing potential of this regional anesthesia technique. This finding is clinically relevant given the growing emphasis on opioid-sparing analgesia strategies in perioperative care. The combination of a superior trunk block with a superficial cervical plexus block appears to provide comprehensive and prolonged analgesia for shoulder surgery, potentially reducing opioid-related adverse effects such as nausea, vomiting, sedation, and respiratory depression.

Neurological safety profile

The neurological safety profile observed in this series was favorable. The absence of persistent neurological symptoms at 24 hours, 7 days, and 14 days is consistent with the literature, which demonstrates that neurological complications after brachial plexus blocks are rare (0.02-0.04%) and generally transient [14-19]. Frost et al., in a secondary analysis of pooled randomized trial data, reported that postoperative neurological symptoms after interscalene block are common in the first 24-48 hours but resolve in the vast majority of cases within two weeks [19].

The transient hoarseness observed in two patients (20%) likely results from local anesthetic spread from the superficial cervical plexus block to the recurrent laryngeal nerve. This complication is well documented in the literature and typically resolves within a few hours, as observed in our series (3 and 9 hours) [2,18]. The absence of Horner syndrome suggests there was no significant spread to the cervical sympathetic chain, possibly related to the superficial cervical plexus block technique used, which deposits the anesthetic in a more superficial plane.

The absence of symptomatic hemidiaphragmatic paralysis is an important finding, consistent with the theoretical advantage of the superior trunk block over the traditional interscalene block. Kim et al. reported an incidence of hemidiaphragmatic paralysis of only 4.8% with a superior trunk block, versus 71.4% with an interscalene block [7]. The local anesthetic volume used in our series (11-14 mL) is within the recommended range to minimize this complication [14-16].

Comparison with international literature

Our findings align with growing international evidence supporting the use of regional anesthesia alone for shoulder surgery. Cohen-Balaloum et al. reported that 87% of 482 patients successfully underwent shoulder arthroscopy under regional anesthesia alone, with superior hemodynamic stability compared to combined approaches (hypotension in 13% vs 57%) [1]. Our hypotension rate of 30% (16.7% in arthroscopies) is intermediate, possibly reflecting differences in hypotension definition or the use of propofol TCI sedation.

PACU length of stay in our series (42.8 minutes for arthroscopies) was comparable to that reported by Cohen-Balaloum et al. (22 minutes for regional anesthesia alone vs 52 minutes for combined anesthesia) [1].

Clinical implications for Brazilian practice

These preliminary results suggest that a change in practice from combined general-regional anesthesia to regional anesthesia alone is feasible in Brazil. Observed advantages include (1) excellent immediate postoperative analgesia (pain 0 at PACU); (2) minimal opioid requirements (90% opioid-free at 24 hours); (3) rapid recovery (PACU time of 51 minutes); (4) high ambulatory procedure rate (70%); (5) favorable safety profile with only minor transient complications; and (6) high patient satisfaction (NPS 10). This approach may be particularly valuable for ambulatory shoulder surgery, where rapid recovery and same-day discharge are priorities, thereby reducing hospital costs and optimizing resources [1].

Technical considerations

The choice of the out-of-plane technique for the superior trunk block, although less common in the literature, offers potential advantages, including better sonoanatomic visualization and lower theoretical risk of injury to the long thoracic and dorsal scapular nerves. The local anesthetic volume used (11-14 mL of 0.5% ropivacaine) is within the recommended range to minimize hemidiaphragmatic paralysis while maintaining analgesic efficacy [14-16].

Sedation with propofol TCI, Eleveld model, allowed precise titration to maintain patient comfort while preserving verbal communication, which is essential for monitoring block adequacy and early detection of complications. The use of the modified Aldrete-Kroulik score ≥9 as a discharge criterion provided a standardized, validated assessment of PACU discharge readiness.

Implementation considerations

Successful adoption of regional-only anesthesia requires: (1) ultrasound expertise - all blocks should be performed under ultrasound guidance to maximize success rates; (2) patient selection and education - appropriate patient selection and thorough preoperative education are essential for patient acceptance, including guidance about the possibility of transient hoarseness; (3) institutional support - commitment from both anesthesiology and surgical teams is necessary; (4) standardised protocols - clear protocols for sedation, block failure management, and conversion to general anesthesia should be established [4].

Limitations

This study has several important limitations. First, the small sample size (n = 10) limits generalizability and precludes statistical inference about comparative efficacy or effectiveness [9,19,20]. This is a feasibility study demonstrating that the technique can be successfully implemented, not a definitive trial proving superiority. Second, the absence of a control group prevents direct comparison with combined general-regional anesthesia in our population. Third, this is a single-center experience with a single anaesthesiologist, which may not reflect outcomes achievable at other Brazilian centers. Fourth, selection bias may have occurred since only patients willing to undergo awake surgery were included. Finally, although a 14-day follow-up captures most neurological complications, rare late complications may not have been detected [17,19].

Future directions

These preliminary findings support the need for a larger prospective cohort study or randomized clinical trial comparing regional anesthesia alone versus combined general-regional anesthesia for shoulder surgery in Brazil. Such a study should include an adequate sample size to detect differences in clinically important outcomes, including hemodynamic stability, recovery metrics, complications, costs, and patient satisfaction. Additionally, investigating optimal local anesthetic regimens (type, concentration, volume) for a superior trunk block in the Brazilian population would be valuable [14-16].

## Conclusions

A superior trunk block combined with a superficial cervical plexus block as sole anesthesia is feasible and safe for shoulder arthroscopy and arthroplasty, with a 0% conversion rate to general anesthesia, a 100% block success rate, excellent postoperative analgesia, minimal opioid requirements (10% rescue opioid use), and high patient satisfaction in this Brazilian case series. The safety profile was favorable, with only transient hoarseness in 20% of cases (resolved within nine hours) and no Horner syndrome, symptomatic hemidiaphragmatic paralysis, or persistent neurological symptoms at 14-day follow-up. These preliminary findings suggest that a change in practice from routine combined general-regional anesthesia to regional anesthesia alone may be achievable in Brazil. However, larger studies are needed to confirm these results and establish optimal protocols.
